# Protein kinase D deficiency induces a senescence-like phenotype in β-cells and improves glucose and insulin tolerance under high-fat diet conditions

**DOI:** 10.1016/j.molmet.2025.102297

**Published:** 2025-12-03

**Authors:** Wolfgang S. Lieb, Carlos O. Oueslati Morales, Kornelia Ellwanger, Claudia Koch, Sylke Lutz, Stephan A. Eisler, Annika M. Möller, Veronika Leiss, Angelika Hausser

**Affiliations:** 1 Institute of Cell Biology and Immunology, University of Stuttgart, 70569, Stuttgart, Germany; 2 Stuttgart Research Center Systems Biology, University of Stuttgart, 70569, Stuttgart, Germany; 3 Department of Pharmacology and Experimental Therapy, Institute for Experimental and Clinical Pharmacology and Toxicology, Interfaculty Center for Pharmacogenomics and Drug Research, Eberhard Karls University Tübingen, 72074, Tübingen, Germany

**Keywords:** PKD, HFD, Aging, Diabetes, β-cell, Senescence

## Abstract

Insulin secretion from pancreatic β-cells is essential for maintaining glucose homeostasis and preventing type 2 diabetes, a condition closely associated with aging. Although previous studies in mice have shown that both basal and glucose-stimulated insulin secretion increase with age, the underlying mechanisms remained poorly understood. In this study, we identify protein kinase D (PKD) as a critical regulator of β-cell function during aging through its control of cellular senescence. Using β-cell–specific expression of dominant-negative PKDkd-EGFP and the selective PKD inhibitor CRT0066101, we demonstrate that inhibition of PKD activity in mature adult mice induced a senescent-like β-cell phenotype characterized by enlarged cell size and elevated β-galactosidase activity. These changes were associated with decreased expression of the antioxidant enzyme superoxide dismutase 2 and increased levels of reactive oxygen species. Surprisingly, despite promoting a senescent-like phenotype, PKD inhibition significantly improved glucose tolerance, enhanced glucose-stimulated insulin secretion, and protected against high-fat diet–induced glucose and insulin intolerance. These findings highlight the importance of PKD in preserving β-cell function under aging and metabolic stress conditions.

## Highlights

• PKD inhibition induces hallmarks associated with β-cell senescence.
• PKD loss improves GSIS and glucose tolerance in aged mice.
• PKD loss lowers SOD2 and raises ROS, linking it to redox control in β-cells.
• Inhibition of PKD Improves Glucose Homeostasis under HFD conditions.

## Graphical abstract

**Figure:**
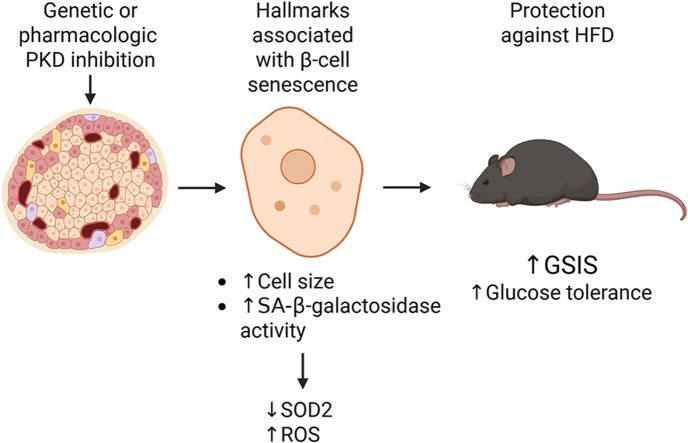

## 1 Introduction

Aging and obesity are major risk factors for the development of type 2 diabetes (T2D) and are closely associated with impaired glucose homeostasis [[1], [2], [3]]. During aging, pancreatic β-cells lose the ability to proliferate in response to higher metabolic demands [4,5]. Consequently, the regenerative potential of β-cells declines markedly in both mice and humans [[6], [7], [8]]. This results in a reduction in functional β-cell mass that contributes to dysregulated insulin secretion, reduced glucose tolerance, and an increased risk of T2D at later stages of life [9]. A key factor underlying the loss of replicative capacity in β-cells is the cyclin-dependent kinase (CDK) inhibitor p16^Ink4a^ (hereafter referred to as p16), a master regulator of cellular senescence. p16 expression increases with age in mouse islets and predisposes animals to diabetes [8,10,11]. Additionally, elevated circulating free fatty acids, such as those induced by a high-fat diet (HFD), suppress β-cell proliferation by inducing p16 expression [12]. Although increased p16 expression is known to limit β-cell regenerative potential, it remained unclear whether this also results in bona fide cellular senescence, and whether such senescent β-cells remain functionally active. Surprisingly, a study demonstrated that p16 promotes β-cell senescence during physiological aging and enhances glucose-stimulated insulin secretion (GSIS) [13]. This finding suggests that p16-dependent β-cell senescence may act as a compensatory mechanism to preserve glucose homeostasis despite a reduced β-cell regenerative capacity. However, the signaling pathways that govern β-cell senescence during aging and their impact on β-cell function remain poorly understood.

The protein kinase D (PKD) family comprises three isoforms, PKD1, PKD2, and PKD3, best known for their roles in dynamic actin remodeling and regulation of vesicle fission at the Trans-Golgi Network (TGN) [14,15]. In the pancreas, PKD isoforms are differentially expressed: PKD3 is predominantly found in exocrine cells of both mouse and human pancreas, whereas PKD1 and PKD2 are more abundantly expressed in the pancreatic islets [16]. Among these, PKD1 has been most extensively studied in pancreatic β-cells. It has been shown to protect against starvation-induced lysosomal degradation of nascent insulin granules both *in vitro* and *in vivo*, thereby protecting β-cell function under stress conditions [17,18]. Consequently, pharmacological inhibition of PKD impairs insulin secretion and accelerates the onset of T2D, whereas PKD1 activation delays T2D onset in obese mice [18]. This supports the finding that PKD1 is essential for the compensatory enhancement of GSIS in response to a HFD *in vivo* [19]. In the INS-1 rat insulinoma β-cell line, PKD1 promotes insulin granule biogenesis and secretion at the TGN, a process negatively regulated by p38δ [20; 21]. However, β-cell–specific PKD1 knockout mice do not display significant defects in β-cell function under physiological conditions, leaving its role in healthy β-cells unresolved [19]. In contrast, global PKD2 deficiency in mice leads to elevated basal and glucose-induced insulin secretion. This hyperinsulinemia contributes to the progressive development of insulin resistance and obesity, a phenotype linked to increased β-cell Ca^2+^ influx [22]. These findings point to isoform-specific and context-dependent roles of PKD in the regulation of β-cell function and systemic glucose homeostasis.

Using *in vivo* and *ex vivo* approaches, we identified a novel function for PKD in regulating β-cell function. Our data reveal that PKD isoform expression in mouse islets is age-dependent. Moreover, expression of dominant-negative PKDkd-EGFP or pharmacological inhibition of PKD activity in mature adult mice induces hallmarks associated with β-cell senescence, improves GSIS and glucose tolerance, and confers protection against HFD–induced glucose and insulin intolerance. These findings underscore the importance of PKD in maintaining β-cell function not only during healthy aging, but also in the context of diet-induced insulin resistance.

## 2 Results

### 2.1 PKD expression is age-dependent in mouse pancreatic islets

**Figure 1:**
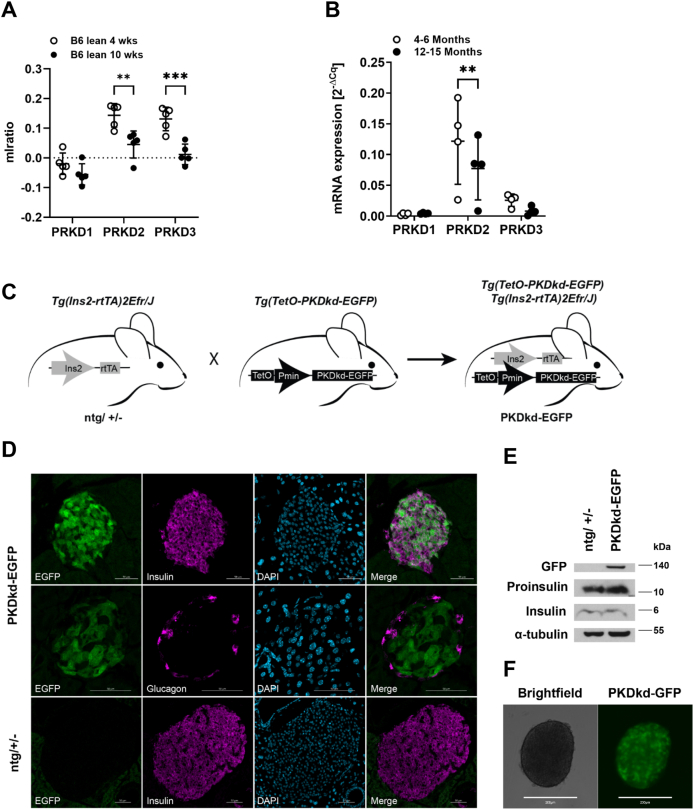
**PKD expression decreases with age in pancreatic β-cells**. **(A)** mRNA expression levels of *PRKD1, 2 and 3* in pancreatic islets from 4- and 10-week-old B6 lean mice. Data are presented as the ratio of mean log_10_ intensity (mlratio), as described in [23] (mean ± SD; *n* = 5). Statistical analysis by two-way ANOVA with Sidák's multiple comparisons test. **(B)** qPCR analysis of *PRKD1, 2 and 3* in isolated islets. Data are presented as mean ± SD; *n* = 4. Statistical analysis by two-way ANOVA with Sidák's multiple comparisons test. **(C)** Breeding scheme for generation of functional PKD knockout mice. The offspring express PKDkd-EGFP in a time-dependent (Tet-On system) and β-cell–specific (*Ins2* promoter) manner. **(D)** Immunofluorescence staining showing selective PKDkd-EGFP expression in β-cells of doxycycline-treated transgenic mice. Pancreatic sections were stained for insulin and glucagon. Images are maximum intensity projections and show EGFP (green), insulin (magenta), glucagon (magenta), and nuclei (DAPI, blue). Scale bar: 50 μm. **(E)** Western blot analysis of isolated islets. Membranes were probed with the indicated antibodies; α-tubulin served as a loading control. **(F)** Microscopy of isolated islets from doxycycline-treated PKDkd-EGFP mice. Left: brightfield; right: EGFP channel. Scale bar: 200 μm.

**Figure S1:**
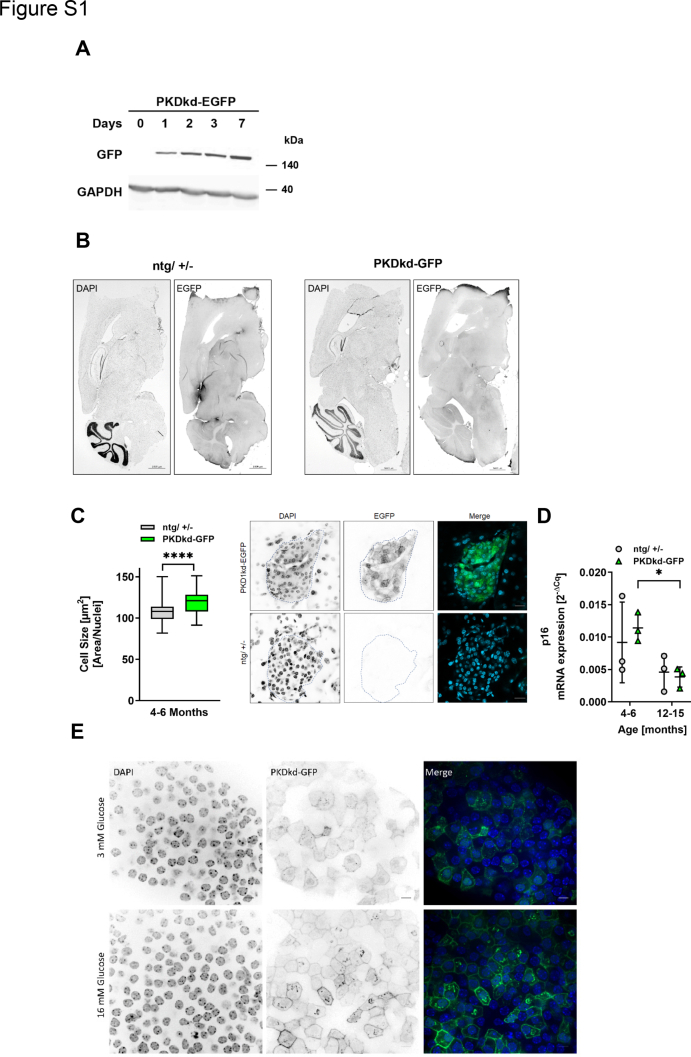
**PKD inhibition increases β-cell size. Related to**Figure 1, Figure 2. **(A)** Western blot analysis of PKDkd-EGFP expression in pancreas tissue following doxycycline administration. Mice received doxycycline in drinking water for the indicated durations. Protein extracts from lysed pancreata were analyzed by immunoblotting using the indicated antibodies, and detected with the Odyssey Scanner. n = 1. **(B)** Brain sections from control (ntg/+/−) and PKDkd-EGFP mice after one week of doxycycline treatment. Brains were fixed in 4% PFA, cryosectioned (30 μm), stained with DAPI, and imaged using a Cell Observer Z.1 microscope. Images are shown as stitched inverted grayscale tiles. Scale bar: 500 μm. **(C)** Quantification of β-cell size. Cryosectioned and stained pancreatic tissue was analyzed by calculating the islet cross-sectional area divided by nuclei number. Right: Representative islets stained with DAPI (blue) and GFP (green). Dashed line denotes the islet boundary. Scale bar: 200 μm. Left: Data are shown as box-and-whiskers plots, with center lines indicating the median and whiskers representing minimum and maximum values; n = 64 (ntg/ +/−), n = 65 (PKDkd-EGFP). Statistical comparison by unpaired, two-tailed *t*-test. **(D)** qPCR analysis of p16 expression in islets. Data are presented as mean ± SD; *n* = 3. **(E)** Glucose stimulation experiment in isolated islets. Islets were incubated in 3 mM glucose for 60 min, followed by fixation or an additional 60 min in 16 mM glucose. Islets were then fixed, stained with DAPI, and imaged using confocal microscopy. Shown is a single z-plane in inverted grayscale. Merge: EGFP (green), DAPI (blue). Scale bar: 10 μm.

All three PKD isoforms are expressed in mouse pancreatic islets with *PRKD1* mRNA expression being the lowest [23]. Analysis of the published dataset further revealed that *PRKD2* and *PRKD3* mRNA levels significantly decreased in 10-week-old lean B6 mice compared to 4-week-old lean B6 mice while *PRKD1* mRNA levels remained unchanged (Figure 1A). To further corroborate this finding, we investigated the expression of the three PKD isoforms in mouse islets isolated from mature adult (4–6 month) and middle-aged (12–15 month) B6 mice [24] as the recovery and plasticity of islet cells decreases once they reach 1-year of age [25]. The analysis showed that compared to *PRKD2* and *PRKD3*, *PRKD1* was expressed at low levels in both, mature adult and middle-aged islets, which is in line with the published results [23]. Strikingly, during aging, the expression of both *PRKD2* and *PRKD3* strongly declined albeit only significant for *PRKD2* (p = 0.09 for *PRKD3*). However, the expression of *PRKD1* remained low and did not change (p = 0.9951), indicating a potential link between β-cell aging and PKD2/3 function (Figure 1B). To address this, we generated transgenic mice with inducible expression of a kinase dead PKD (referred to as PKDkd) EGFP protein [[26], [27], [28]]. PKDkd acts dominant-negative on all three PKD isoforms thereby inducing a functional PKD knockout [29,30]. To express PKDkd–EGFP in an inducible and conditional manner, we used the tetracycline-dependent gene expression system [31]. In this system, the tet activator protein (rtTA) is constitutively expressed under the control of the activator transgene. In the presence of the tetracycline analogue doxycycline, the rtTA protein binds to the TRE (tetracycline responsive promoter element), inducing the expression of the transgene. To analyze the role of PKD in pancreatic β-cells we generated double transgenic mice in which the doxycyline-inducible expression of PKDkd-EGFP is under the control of the rat insulin 2 (*Ins2*) promoter (Figure 1C). PKDkd-EGFP expression was detectable at day one after doxycycline treatment and constantly increased over time, reaching its highest expression after one week (Figure S1A). Upon one week of doxycycline administration via drinking water, we observed an expression of PKDkd-EGFP restricted to the islets of Langerhans. Staining of insulin and glucagon in pancreatic tissue revealed a β-cell specific expression of the transgene, which was not observed in glucagon-positive α-cells (Figure 1D). Importantly, the expression of PKDkd-EGFP alone did not interfere with insulin or proinsulin levels compared to doxycycline-treated control mice (Figure 1E) and remained stable in isolated islets (Figure 1F). The rat *Ins2* promoter additionally shows activity in the brain, however, administration of doxycycline via the drinking water is insufficient for the drug to pass the blood–brain barrier and induce transgene expression in this organ [[32], [33], [34]]. Consequently, upon one week of doxycycline administration via the drinking water we observed no PKDkd-EGFP expression in the brain (Figure S1B).

### 2.2 PKDkd-EGFP expression promotes a senescent-like phenotype in mature adult β-cells

**Figure 2:**
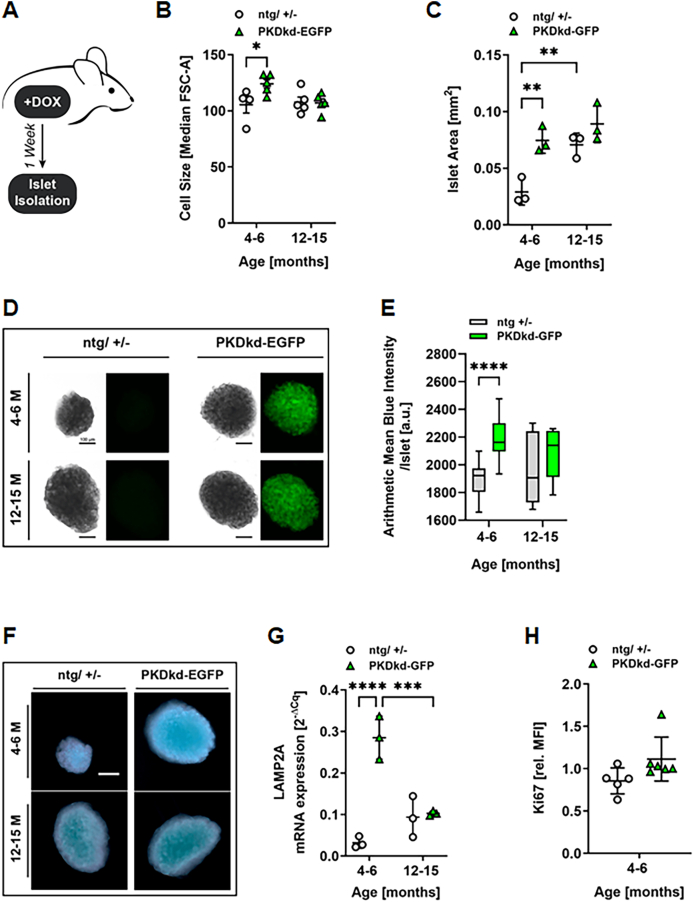
**PKDkd-EGFP expression promotes a senescent-likephenotype in pancreatic β-cells. (A)** Experimental workflow of 7-day doxycycline treatment in PKDkd-EGFP mice. **(B)** Flow cytometry-based analysis of β-cell size in dispersed islet cells. Data are presented as median FSC-A ± SD; *n* = 4–5. **(C)** Islet area quantification. Ten islets per mouse were analyzed using ImageJ. Data are shown as mean islet area (mm^2^) ± SD; *n* = 3. Statistical analysis by two-way ANOVA with Sidák's multiple comparisons test. **(D)** Representative brightfield and EGFP images of islets analyzed in (C). Scale bar: 100 μm. **(E) (E)** SA-β-galactosidase staining of isolated islets. Blue staining intensity was quantified using ZEN software. Data are shown as box-and-whiskers plots; center lines represent medians, whiskers indicate min and max; *n* = 24, 24, 7, 9 (groups as indicated). Statistical analysis by two-way ANOVA with uncorrected Fisher's LSD test. **(F)** Representative images of SA-β-galactosidase-stained islets. Scale bar: 100 μm. **(G)** qPCR analysis of *Lamp2a* expression in islets. Data are presented as mean ± SD; *n* = 3. Statistical analysis by two-way ANOVA with Sidák's multiple comparisons test. **(H)** Proliferation analysis of islet cells by Ki67 staining Quantification of Ki67+ cells by flow cytometry, presented as mean relative MFI ± SD; n = 5–6. Statistical analysis by t-test.

During aging, the decline in replicative capacity of β-cells coincides with increased promoter methylation and decreased expression of cell cycle regulators. Healthy β-cell aging is thus associated with a reduction in β-cell mass and induction of cellular senescence [9]. The observed downregulation of *PRKD2* and *PRKD3* gene expression in islets during aging suggests a potential connection of the kinases with the regulation of cellular senescence. To address this, mature adult and middle-aged PKDkd-EGFP and control (ntg/+/−) mice received 2 mg/ml doxycycline on seven consecutive days, followed by islet isolation and immediate analysis for hallmarks associated with senescence such as increased cell size and senescence-associated (SA)-β-galactosidase activity (Figure 2A). When comparing middle-aged and mature adult control mice, we observed that β-cell and islet size (Figure 2B–D) were markedly increased. SA-β-galactosidase activity was slightly enhanced in middle-aged control mice compared to mature adult control mice (Figure 2E,F). Strikingly, in PKDkd-EGFP mature adult mice β-cell and islet size, as well as SA-β-galactosidase activity, were significantly increased to levels comparable to those of middle-aged control mice (Figure 2B–F, Figure S1C). In middle-aged mice, PKDkd-EGFP expression did not significantly affect β-cell or islet size and SA-β-galactosidase activity (Figure 2B–F). In line with these findings, we observed significantly increased gene expression of the lysosomal protein *Lamp2A* in mature adult, but not middle-aged, PKDkd-EGFP mice compared to age-matched control mice (Figure 2G). A similar trend was observed for *p16*, albeit not significant (Figure S1D). In mice, adult β-cells are long-lived and divide only rarely, with replication occurring in fewer than 1% of cells per day [35]. Consistent with this, we found low Ki67 levels in islets from middle-aged mice, however, with no significant change upon PKDkd-EGFP expression (Figure 2H). Thus, our results clearly connect PKD activity with hallmarks associated with cellular senescence.

### 2.3 Functional PKD knockout improves glucose tolerance and GSIS

It is well known that during healthy aging of mice β-cell functionality changes. However, while some reports observed an increase in basal insulin secretion and impaired GSIS with age [36; 37], others reported improved β-cell functionality [13; 38; 39]. As PKDkd-EGFP expression in mature adult mice induced hallmarks associated with cellular senescence (Figure 2D,F), we analyzed whether PKDkd-EGFP mature adult mice show an improved β-cell function and therefore performed glucose tolerance test (GTT), insulin tolerance test (ITT) as well as GSIS. After one week of doxycycline treatment, mice were starved for 16 h, followed by an intraperitoneal injection of glucose. Blood samples were collected from the tail tip and blood glucose levels were measured. In line with the induction of a senescence-like phenotype, mature adult PKDkd-EGFP expressing mice exhibited significantly improved glucose tolerance (Figure 3A,B), while insulin tolerance remained unaffected (Figure 3C,D). In middle-aged mice, glucose and insulin tolerance were strongly diminished independently of PKDkd-EGFP expression (Figure 3A–D).

**Figure 3:**
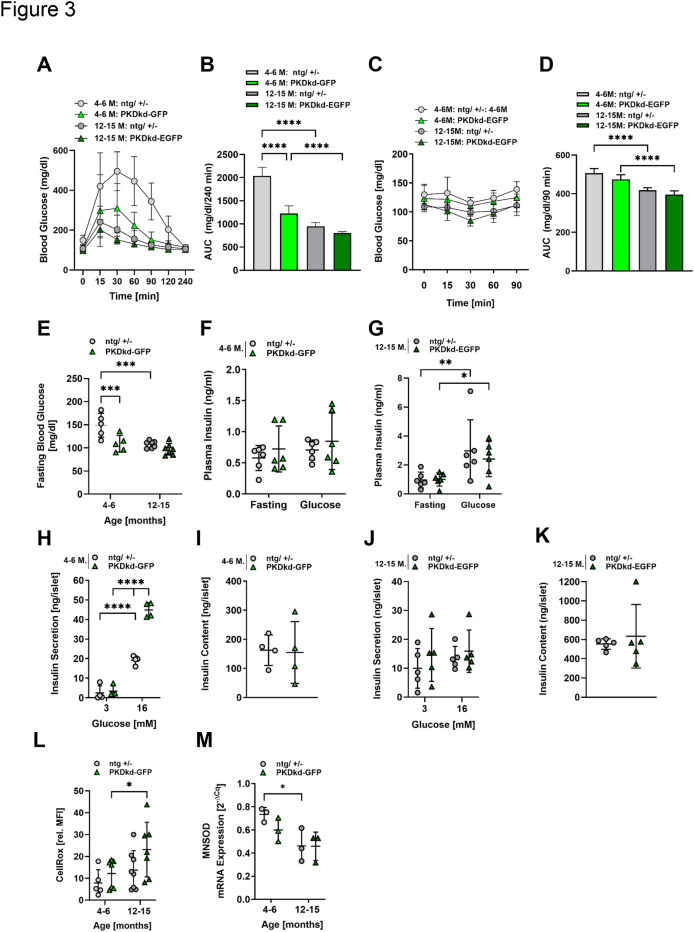
**PKDkd-EGFP expression improves glucose tolerance *in vivo* and enhances GSIS in isolated islets. (A)** Glucose tolerance test (GTT) in 4–6-month-old and 12–15-month-old ntg/+/− (light and dark grey circles) and PKDkd-EGFP (light and dark green triangles) mice. Blood glucose levels were measured before and after i.p. injection of 2 mg/g body weight glucose. Data are presented as mean ± SD; *n* = 5 (4–6 months), *n* = 7 (12–15 months). **(B)** Area under curve (AUC) analysis of GTT data from (A). Data are shown as mean ± SD. Statistical analysis by one-way ANOVA with Tukey's multiple comparisons test. **(C)** Insulin tolerance test (ITT) in 4–6-month-old and 12–15-month-old mice. Blood glucose was measured at indicated time points following insulin injection. Data are shown as mean ± SD; *n* = 5–6. **(D)** AUC analysis of ITT data from (C). Data are presented as mean ± SD. Statistical analysis by one-way ANOVA with Tukey's multiple comparisons test. **(E)** Fasting blood glucose levels following 16 h fast. Data are shown as mean ± SD; *n* = 5–7. **(F, G)** Plasma insulin levels before and 15 min after glucose injection in 4–6-month-old **(F)** and 12–15-month-old **(G)** mice. Mice were fasted overnight and challenged with glucose. Data are presented as mean ± SD; *n* = 4–6. **(H)** Ex vivo GSIS in islets from 4–6-month-old mice. Data are shown as mean insulin secretion per islet (ng/islet) ± SD; *n* = 4. **(I)** Total insulin content of islets from 4–6-month-old mice following extraction in acidic ethanol. Insulin was quantified by ELISA. Data are shown as mean ± SD; *n* = 4. **(J)** Ex vivo GSIS in islets from 12 to 15-month-old mice. Data are shown as mean insulin secretion per islet (ng/islet) ± SD; *n* = 5. **(K)** Total insulin content of islets from 12 to 15-month-old mice, as in (I). Data are presented as mean ± SD; *n* = 5. **(L)** ROS levels in dispersed islet cells, measured via flow cytometry following CellRox staining. Data are presented as mean relative MFI ± SD; *n* = 6–8. **(M)** qPCR analysis of *MNSOD* expression in islets. Data are presented as mean ± SD; *n* = 3. Statistical analysis in (E–M) was performed using two-way ANOVA with Sidák's multiple comparisons test.

In line with the literature [40], fasting blood glucose levels were reduced in middle-aged mice compared to mature-adult mice (Figure 3E). Interestingly, the fasting blood glucose levels in mature adult PKDkd-EGFP expressing mice were significantly reduced when compared to control mice while no difference in middle-aged mice was observed (Figure 3E). Moreover, the *in vivo* GSIS showed slightly elevated plasma insulin levels in mature adult PKDkd-EGFP expressing mice, before glucose injection and 15 min afterwards when compared to control mice of the same age (Figure 3F). In middle-aged mice of both genotypes, plasma insulin levels were in general increased compared to mature adult mice, however, expression of PKDkd-EGFP had no effect (Figure 3G). These results suggest that the expression of PKDkd-EGFP increases insulin secretion into the blood stream in mature adult mice. To test this hypothesis, islets of control and PKDkd-EGFP mature adult mice were isolated and *ex vivo* GSIS experiments were conducted. Islets from the two groups of mice secreted similar levels of insulin after incubation in low glucose, which significantly increased in high glucose (Figure 3H). However, PKDkd-EGFP expression significantly enhanced insulin secretion in mature adult islets incubated in high-glucose medium compared to matched control islets (Figure 3H), whereas this was not observed in middle-aged islets (Figure 3J). Notably, total insulin levels remained unchanged compared to control mice of the same age (Figure 3I,K) indicating that the increased secretion was not due to increased insulin expression. Microscopic analysis revealed that under low-glucose conditions, PKDkd-EGFP displayed a diffuse cytoplasmic distribution with slight enrichment at the plasma membrane and localization to discrete cytoplasmic puncta. Upon high-glucose stimulation, PKDkd-EGFP was recruited to the plasma membrane and a perinuclear region, likely corresponding to the TGN, in pancreatic β-cells (Figure S1E). This translocation is consistent with glucose-induced production of diacylglycerol (DAG) [41], which is known to recruit PKD to membranes via its C1 domain [42]. Moreover, in line with the proposed increase in basal insulin secretion during aging [36], insulin secretion and insulin content of middle-aged islets under low glucose were strongly enhanced compared to islets of mature adult mice (Figure 3H–K).

Reactive oxygen species (ROS) have been described to promote senescence in human epidermal keratinocytes [43] and mouse fibroblasts [44]. Notably, mitochondrial ROS activates PKD, which initiates the NFκB pathway and facilitates the expression of the ROS scavenging protein SOD2, also known as MNSOD [45]. To address whether the expression of dominant-negative PKDkd affects ROS levels in β-cells, we analysed the expression of *MNSOD* by qPCR and examined the ROS content in isolated islets by flow cytometry. As previously reported [46], *MNSOD* expression declines with age (Figure 3M). Consistent with this observation, we also detected increased ROS levels during aging (Figure 3L). Notably, in islets expressing PKDkd-EGFP from mature adult mice, MNSOD gene expression was significantly downregulated, while ROS levels were upregulated compared to age-matched control mice (Figure 3M,L). PKDkd-EGFP expression had no effect on *MNSOD* expression (Figure 3M), while ROS levels were also upregulated in middle-aged mice (Figure 3L). These findings demonstrate that the expression of dominant-negative PKDkd-EGFP reduced *MNSOD* gene expression and facilitated ROS upregulation in mature adult mice.

### 2.4 Pharmacological inhibition of PKD induces a senescence-like phenotype in β-cells and improves glucose tolerance

To validate our findings in a more clinically relevant setting and to exclude off-target effects by the PKDkd-EGFP transgene, we used the selective pharmacological pan-inhibitor of all PKD isoforms, CRT0066101 [47], in mature adult wild-type mice. Inhibitor or carrier control was given once daily p.o. for seven consecutive days. Afterwards, we analyzed glucose and insulin tolerance (Figure 4A). In line with our previous results obtained in mature adult PKDkd-EGFP mice, CRT0066101 treatment significantly improved glucose tolerance (Figure 4B,C) and insulin tolerance (Figure 4D,E). We also observed that pharmacological inhibition of PKD significantly enhanced GSIS (Figure 4F). Additionally, β-cell size was also increased (Figure 4G), and SA-β-galactosidase expression was elevated (Figure 4H,I). Although not all of these differences reached statistical significance, the consistent trends across all three phenotypes suggest a shift toward a senescent-like β-cell state. Finally, pharmacological inhibition of PKD reduced *MNSOD* expression and modestly increased ROS levels in β-cells (Figure 4J,K), thereby validating our findings in PKDkd-EGFP–expressing mice.

**Figure 4:**
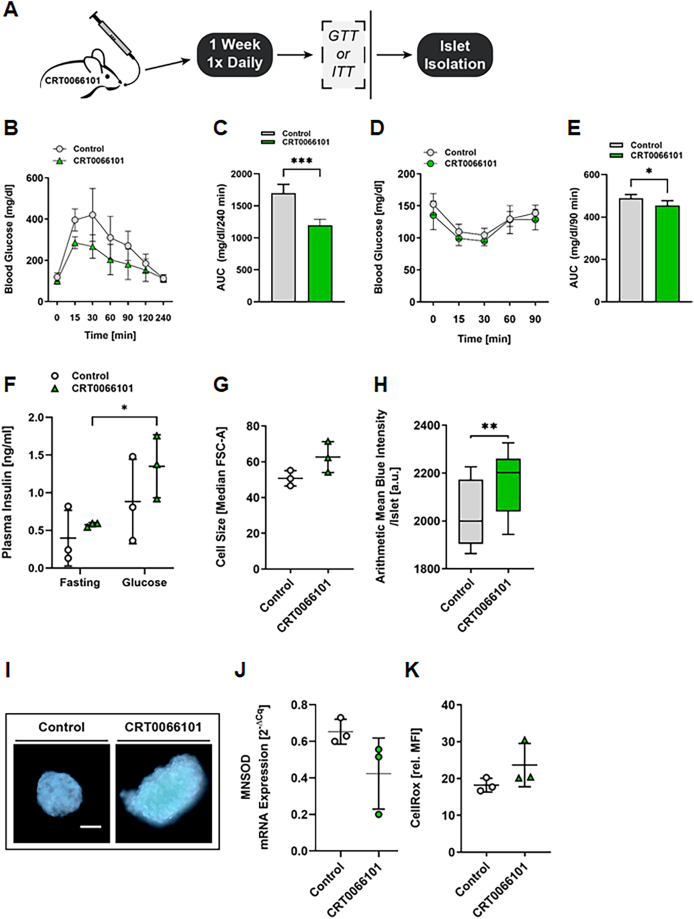
**CRT0066101 treatment improves glucose tolerance and induces a senescent-likephenotype in pancreatic islets. (A)** Experimental workflow of CRT0066101 treatment in 4–6-month-old WT mice. **(B)** GTT following one week of CRT0066101 treatment. Blood glucose levels were measured at indicated time points after glucose injection. Data are presented as mean ± SD; *n* = 5. **(C)** AUC analysis of GTT data from (B). Data are shown as mean ± SD. Statistical comparison by unpaired, two-tailed *t*-test. **(D)** ITT following one week of treatment. Blood glucose levels were measured at indicated time points after insulin injection. Data are presented as mean ± SD; *n* = 5–6. **(E)** AUC analysis of ITT data from (D). Data are shown as mean ± SD. Statistical comparison by unpaired, two-tailed *t*-test. **(F)** Plasma insulin levels before and 15 min after glucose injection, measured by ELISA. Mice were fasted overnight prior to glucose challenge. Data are presented as mean ± SD; *n* = 3. Statistical analysis by two-way ANOVA with Šidák's multiple comparisons test. **(G)** Flow cytometry-based cell size analysis of dispersed islet cells (FSC-A). Data are shown as median ± SD; *n* = 3. **(H)** SA-β-galactosidase staining of isolated islets following treatment. Data represent quantification of blue staining intensity, shown as box-and-whiskers plots; center lines denote medians, whiskers indicate min and max; *n* = 15 (control), 18 (treated). **(I)** Representative images of SA-β-galactosidase-stained islets. Scale bar: 100 μm. **(J)** qPCR analysis of *MNSOD* expression in islets. Data are presented as mean ± SD; *n* = 3. **(K)** Flow cytometry-based ROS quantification in islet cells stained with CellRox. Data are shown as mean relative MFI ± SD; *n* = 3. Statistical comparisons in (G–L) were performed using unpaired, two-tailed *t*-tests.

### 2.5 PKDkd-EGFP expression protects from high-fat-diet-induced insulin resistance

**Figure 5:**
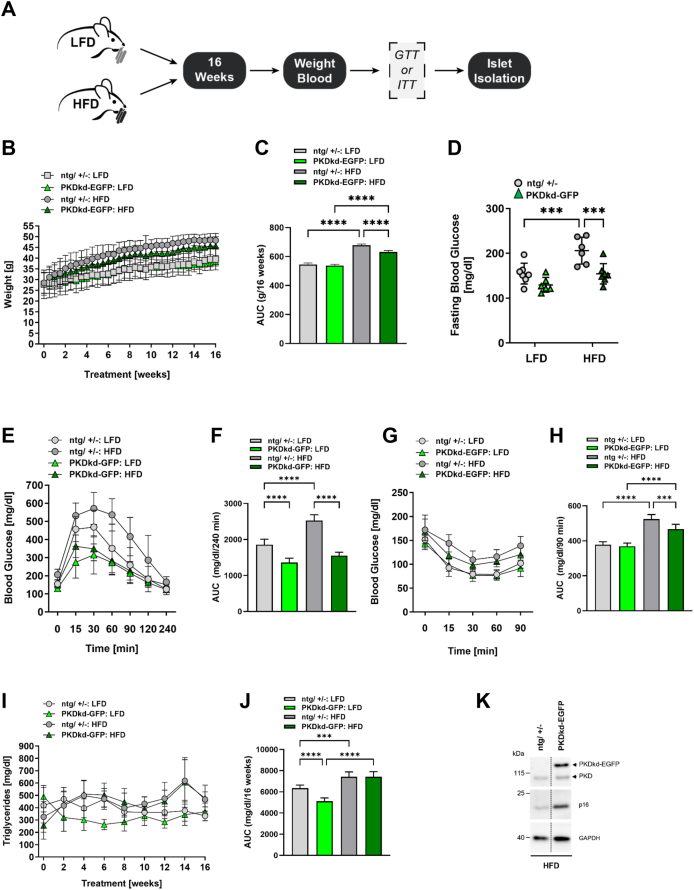
**PKDkd-EGFP expression improves glucose tolerance and protects against HFD-induced insulin resistance. (A)** Experimental workflow of LFD and HFD feeding in transgenic mice expressing PKDkd-EGFP. **(B)** Body weight of LFD- and HFD-fed mice over time. Data are presented as mean ± SD; n = 7. **(C)** AUC analysis of body weight data from (B). Data are shown as mean ± SD. **(D)** Fasting blood glucose levels after 16 weeks of diet. Data are mean ± SD; n = 6–7. Statistical analysis by two-way ANOVA with Šidák's multiple comparisons test. **(E)** GTT in LFD- and HFD-fed mice. Blood glucose was measured at indicated time points following glucose injection. Data are mean ± SD; n = 6–7. **(F)** AUC of GTT data from (E). Data are shown as mean ± SD. **(G)** ITT in LFD- and HFD-fed mice. Blood glucose was measured at indicated time points following insulin injection. Data are mean ± SD; n = 5–7. **(H)** AUC of ITT data from (G). Data are shown as mean ± SD. **(I)** Plasma triglyceride levels in LFD- and HFD-fed mice. Data are mean ± SD; n = 7. **(J)** AUC of triglyceride data from (I). Data are shown as mean ± SD. **(K)** Immunoblot analysis of islets isolated from HFD mice. Membranes were probed with indicated antibodies; PKD expression was detected with a PKD-specific antibody. GAPDH served as a loading control. Detection of membranes was done at the Amersham imager 600 device. The vertical line indicates a cropped lane from the same blot. Statistical analysis in (C, F, H, J) was performed using one-way ANOVA with Šidák's multiple comparisons test.

**Figure S2:**
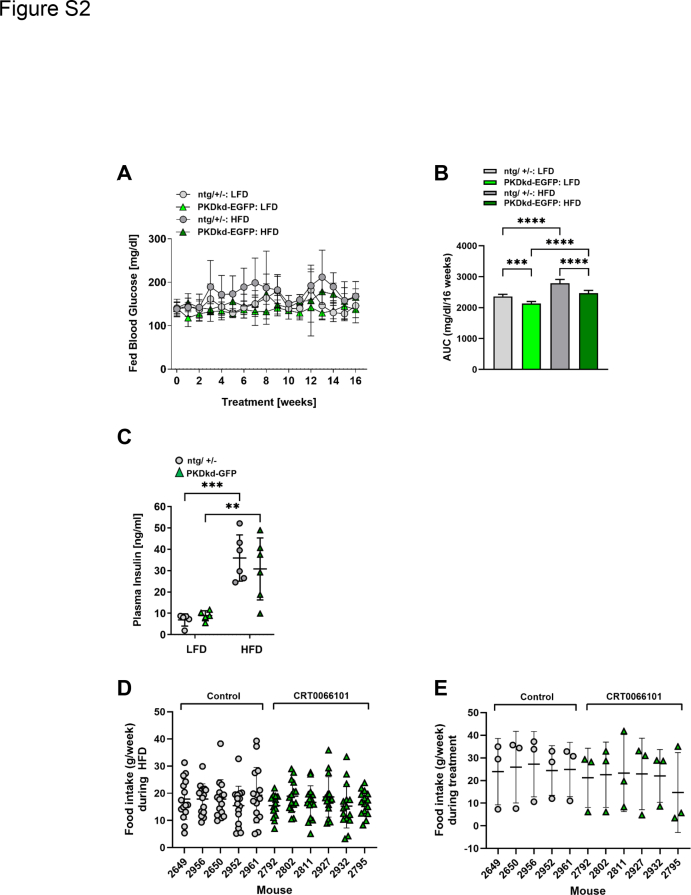
**PKD inhibition improves fed blood glucose levels. Related to**Figure 5, Figure 6**. (A)** Weekly fed blood glucose measurements during the HFD period in LFD- and HFD-fed mice. Data are presented as mean ± SD; *n* = 7 per group. (**B)** AUC analysis of data from (A). Data are shown as mean ± SD. Statistical analysis by one-way ANOVA with Sidák's multiple comparisons test. **(C)** Plasma insulin levels of HFD and LFD-fed mice at week 16. Data are presented as mean ± SD; *n* = 5–6 per group. Statistical analysis by two-way ANOVA with Sidák's multiple comparisons test. **(D, E)** Weekly food intake per mouse during the 16-week HFD phase **(C)** and the 3-week treatment phase **(D)**. Data are presented as mean ± SD; *n* = 5–6 per group.

To test the improved glucose tolerance in a disease setting, we placed young (2 M old) PKDkd-EGFP expressing and control (ntg/+/−) mice on HFD. The HFD model is a widely accepted model for lipid-induced insulin resistance [48]. Mice fed with a low-fat purified diet (LFD) containing the same ingredients with a closely matched composition to the high-fat formula were used as control groups (Figure 5A). As expected, body weight of HFD mice drastically increased over time, however, body weight gain of PKDkd-EGFP mice was markedly decreased compared to control mice. Body weight gain of mice on LFD, however, was similar for both genotypes (Figure 5B,C). Fed blood glucose levels were elevated in both control and PKDkd-EGFP mice on a HFD compared to their respective LFD counterparts (Figure S2A, B). PKDkd-EGFP mice, whether on LFD or HFD, exhibited significantly lower blood glucose levels compared to the corresponding control groups (Figure S2A, B). After 16 weeks, the fasting blood glucose levels were tested and GTT as well as ITT were performed. Fasting glucose levels were significantly higher in HFD control mice compared to control mice on LFD (Figure 5D). Strikingly, the blood glucose levels of PKDkd-EGFP mice on HFD did not differ from those of PKDkd-EGFP mice on LFD and were significantly decreased compared to control mice on HFD (Figure 5D). Moreover, the glucose tolerance of HFD-fed and LFD-fed PKDkd-EGFP animals was significantly improved compared to control HFD and LFD animals, respectively (Figure 5E,F). HFD-fed control animals displayed a strong insulin resistance, compared to the respective LFD-fed animals (Figure 5G,H). HFD-fed PKDkd-EGFP animals also showed insulin resistance when compared to the LFD-fed PKDkd-EGFP animals. Importantly, HFD-fed PKDkd-EGFP animals showed a partial protection against lipid-induced insulin resistance compared to HFD-fed control animals indicating that the β-cell specific loss of PKD activity improved glucose tolerance and protected, at least in part, from insulin resistance (Figure 5G,H). Consistent with this, plasma insulin levels at week 16 were significantly higher in both control and PKDkd-EGFP mice on HFD compared with LFD-fed mice, reflecting hyperinsulinemia [49], however, the increase was less pronounced in PKDkd-EGFP mice (Figure S2C). HFD increases triglycerides levels in the blood [50], which can be converted to free fatty acids and thereby increase insulin resistance [51,52]. Indeed, blood triglyceride levels were significantly higher in HFD-fed mice compared to LFD-fed mice (Figure 5I,J). In LFD-fed mice, the expression of PKDkd-EGFP significantly reduced the blood triglyceride content compared to control mice (Figure 5I,J).This finding is consistent with the slightly elevated plasma insulin levels observed upon PKDkd-EGFP expression in mature adult mice (Figure 3F). In contrast to body weight gain and glucose and insulin tolerance, the blood triglyceride concentration did not differ between HFD-fed control and PKDkd-EGFP animals (Figure 5I,J) showing that PKDkd-EGFP expression was not sufficient to fully protect from the HFD-induced metabolic syndrome. Consistent with our previous data, islets from HFD-fed PKDkd-EGFP mice contained increased levels of p16 compared to the respective control mice, pointing to the development of a senescence-like phenotype (Figure 5K).

### 2.6 Pharmacological PKD inhibition rescues glucose and insulin tolerance in high-fat diet-fed mice

**Figure 6:**
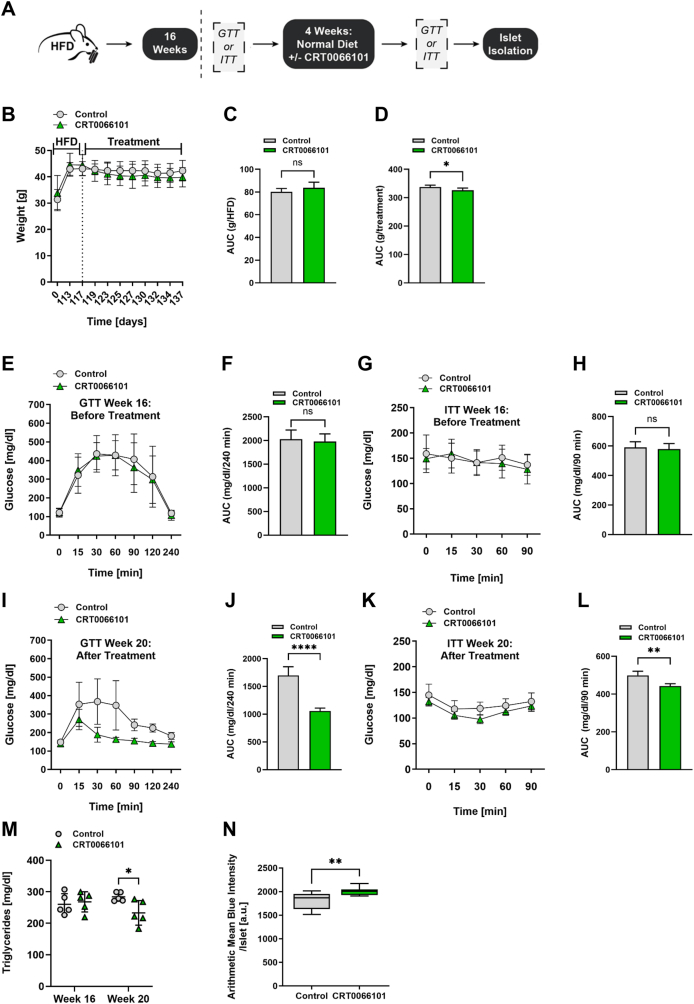
**Pharmacological inhibition of PKD with CRT0066101 improves metabolic function in HFD-fed pre-diabetic mice. (A)** Experimental workflow outlining HFD feeding and subsequent treatment with the PKD inhibitor CRT0066101 in wild-type mice. **(B)** Body weight measurements before and after CRT0066101 treatment. Data are presented as mean ± SD; n = 5–6 per group. **(C, D)** AUC analysis of body weight during the HFD phase (C) and during treatment (D). **(E, G)** GTT (E) and ITT (G) performed prior to treatment. Blood glucose levels were assessed at indicated time points following glucose or insulin administration. Data are shown as mean ± SD; n = 5–6. **(F, H)** AUC analysis of data from (E) and (G), respectively. Data are presented as mean ± SD. **(I, K)** GTT (I) and ITT (K) following CRT0066101 treatment. Data are shown as mean ± SD; n = 5–6. **(J, L)** AUC analysis of data from (I) and (K), respectively. Data are presented as mean ± SD. **(M)** Plasma triglyceride levels at week 16 (pre-treatment) and week 20 (post-treatment). Data are presented as mean ± SD; n = 5. **(N)** SA-β-galactosidase staining of pancreatic islets isolated from 4–6-month-old wild-type mice following treatment. Blue staining intensity was quantified using ZEN software. Data are shown as box-and-whiskers plots, with center lines indicating the median and whiskers representing minimum and maximum values; n = 9 (control), 12 (treated). Statistical significance in (C, D, F, H, J, L, M, N) was assessed using unpaired, two-tailed t-tests.

As the expression of PKDkd increased glucose sensitivity and decreased insulin resistance in a HFD model, we tested if pharmacological PKD inhibition would be beneficial in an already existing HFD-induced insulin and glucose intolerance setting as well (Figure 6A). Thus, we put mature-adult wild-type mice on HFD for 16 weeks (Figure 6B) and performed ITT and GTT. As expected, we did not detect any differences in weight gain, glucose and insulin tolerance between groups before treatment with the carrier or CRT006101 (Figure 6C, E-H). After HFD, mice received a normal diet and were concurrently treated with either CRT0066101 or the carrier once a day for weeks 17–19, again followed by GTT and ITT (Figure 6A,B). During the treatment phase, CRT0066101-treated mice experienced body weight loss compared to control mice (Figure 6B,D), despite no differences in food intake (Figures S2D, E). Following treatment, CRT0066101 significantly improved HFD-induced glucose and insulin intolerance (Figure 6I–L). Additionally, PKD inhibition markedly reduced plasma triglyceride levels (Figure 6M). Consistent with our previous findings, SA-β-galactosidase activity was upregulated following PKD inhibition (Figure 6N).

In summary, our data show that both PKDkd-EGFP expression and pharmacological inhibition (CRT0066101 treatment) of PKD induce a senescent-like phenotype in pancreatic β cells associated with enhanced GSIS and protect mice from HFD-induced obesity, glucose intolerance, and insulin resistance.

## 3 Discussion

This study is the first to demonstrate an essential role for PKD in maintaining β-cell function during aging. We found that β-cell-specific expression of dominant-negative PKDkd, or pharmacological inhibition of PKD with CRT0066101, induced hallmarks associated with senescence and improved glucose tolerance in mature adult mice, but not in middle-aged mice. Furthermore, PKDkd-EGFP expression protected mice from HFD-induced insulin and glucose intolerance. Remarkably, CRT0066101 was highly effective in preventing insulin and glucose intolerance in HFD-fed mice.

The function of PKD1 in pancreatic β-cells has been intensively studied in recent years. However, direct examination of PKD1 function in β-cells has been carried out almost exclusively in the rat insulinoma cell line INS-1 [17,20,53]. Other studies have indirectly investigated PKD1 signaling *in vivo*, and have demonstrated that GPR-40, p38MAPK and the M3-Muscarinic receptor regulate this kinase in β-cells [21,53,54]. Up to date, only one study assessed the direct effect of a β-cell specific PKD1 knockout in a mouse model and observed that PKD1 is dispensable for β-cells under basal conditions but is required for the compensatory increase in GSIS in response to HFD [19]. In contrast to other studies [18,21], we observed that PKD2 and PKD3 were expressed at much higher levels than PKD1 in β-cells. We also observed that PKD2 and PKD3 expression decreased in middle-aged animals, which correlates with data on inactivating methylation of the *PRKD2* and *PRKD3* promoter in mouse islets upon aging [38] and further suggests a functional role of PKD2 and PKD3 in the aging process. Therefore, in this study, a PKDkd-EGFP expressing mouse model and the pan-PKD inhibitor CRT were used to block PKD activity in general.

Islets from PKDkd-EGFP mice and CRT0066101 treated mice displayed upregulated *Lamp2A* and p16 expression levels, respectively, in mature adult mice, which correlated with other markers associated with senescence, such as increased cell size, islet area and SA β-galactosidase activity. Interestingly, we did not observe the same effects in middle-aged mice. However, based on our observations that endogenous PKD expression decreases with aging, the dominant-negative effect of PKDkd-EGFP expression may simply lose its effect. Moreover, p16 expression steadily increases with aging [10] and PKDkd-EGFP expression may not further enhance this effect.

To rule out off-target effects of transgenic PKDkd-EGFP expression, we treated wild-type mice with the pan-PKD inhibitor CRT0066101 [55] and analyzed β-cell function. Remarkably, CRT0066101 also induced hallmarks associated with β-cell senescence, along with improved glucose tolerance and enhanced *in vivo* GSIS. In this pharmacological setting, PKD inhibition was not restricted to pancreatic β-cells and thus additional cell types and tissues may contribute to the observed phenotype. For example, adipocyte-specific knockout of PKD1 improved insulin and glucose tolerance *in vivo*, conferred protection against obesity and T2D, and promoted the formation of beige adipocytes [56]. In addition, PKD2 inactivation has been shown to limit intestinal lipid absorption, thereby protecting against diet-induced obesity [57]. The pronounced improvement in glucose tolerance and the reduction of triglyceride levels observed with CRT0066101 treatment thus reflects the effects of systemic PKD inhibition. Consistently, global PKD2 deficiency in 4-week-old mice led to increased fasting insulin levels, decreased fasting glucose levels, and enhanced insulin secretion from β-cells under both basal and glucose-stimulated conditions by 14 weeks of age [22]. These findings align largely with the phenotype observed in our mature adult animals. However, in contrast to our data based on 16-week PKDkd-EGFP expression, Xiao et al. also reported an increase in body weight and blood glucose levels in PKD2 knockout mice [22]. This discrepancy suggests that global PKD2 deficiency, rather than β-cell-specific PKD inhibition, may be required to influence systemic metabolic outcomes such as obesity and insulin resistance.

Our data are consistent with a study by Helman and coworkers, which demonstrated that forced expression of p16 in β-cells from middle-aged mice induced features of senescence, such as increased β-cell size. Notably, p16 expression improved glucose tolerance in middle-aged mice and GSIS increased during aging [13]. Other studies in young (1–6 months) and middle-aged mice (12–18 months) also showed that with aging basal and stimulated insulin secretion increases [36,37,39]. However, there are also studies that contradict our findings. For example, Wang et al. showed in rats that islet size was the same in mature adult (4–5 months) and aged (21–22 months) animals and senescent islets secreted less insulin, compared to adult islets [58]. This suggests that the age (and probably the strain) of the animals has a major influence on the outcome.

We further found that the expression of PKDkd-EGFP and the inhibition of PKD by CRT0066101 decreased *MNSOD* expression and increased ROS levels in islets. Previous studies showed that ROS-induced PKD activity drives the expression of *MNSOD* in HeLa cells [45,59]. *MNSOD* deficiency in mice led to increased mitochondrial oxidative damage and promoted p16-dependent cellular senescence in the skin [60]. Since β-cells have relatively low antioxidant capability and are therefore more susceptible to oxidative stress, enhanced ROS production triggers their senescence by impairing mitochondrial homeostasis [61]. Notably, in aged animals, this decline in mitochondrial function is partially compensated by β-cell plasma membrane hypersensitivity due to reduced K_ATP_ channel conductance, which enhances Ca^2+^ influx and insulin secretion and may be attributed to increased p16 expression [39]. Consistently, PKD2 knockout mice show increased β-cell Ca^2+^ influx [22]. Thus, our results provide a mechanistic link between loss of PKD activity and the induction of a senescent-like phenotype in β-cells via downregulation of *MNSOD* expression and the consequent upregulation of ROS and p16. Whether PKD inhibition also affects K_ATP_ channel conductance and thus glucose responsiveness, especially under high-glucose conditions, remains an intriguing question for future investigations.

We also observed that HFD-fed PKDkd-EGFP mice had reduced body weight and improved glucose and insulin tolerance compared to control mice. At first glance, these findings appear to contradict a previous study in which β-cell-specific knockout of PKD1 exacerbated HFD-induced glucose intolerance [19]. This phenotype was associated with impaired glucose-induced insulin secretion both *in vivo* and *ex vivo* without changes in islet mass [19]. However, that study focused exclusively on PKD1, which is expressed at lower levels in β-cells compared to PKD2 and PKD3, suggesting that the metabolic impact of PKD inhibition may depend on the specific isoform targeted. Because prolonged β-cell senescence deteriorates cellular function followed by β-cell exhaustion and β-cell death [62], the beneficial effects of PKD inhibition, namely increased insulin secretion and improved glucose tolerance, might be restricted.

Assessment of β-cell health and insulin secretory capacity *in vivo*, which would provide direct insight into the impact of PKD activity on functional β-cell mass, was not feasible in the present study and thus represents a limitation in the interpretation of our findings. Nevertheless, our results are consistent with a functional role for PKD in the aging and senescence of pancreatic β-cells and suggest that PKD inhibition may enhance glucose tolerance and glucose-stimulated insulin secretion, thereby helping to protect against insulin intolerance in a HFD mouse model. Together, these findings offer new perspectives on the context-dependent roles of PKD in β-cell biology and raise important questions about how different PKD isoforms integrate metabolic and stress signals to regulate β-cell fate over time.

## 4 Materials and methods

### 4.1 Key resource table

**Table:** 

Reagent or Resource	Source	Identifier
Antibodies		
Anti-α tubulin (DM1A) mouse	Millipore	Cat# 05-829 RRID:AB_310035
Anti-GAPDH (14C10) rabbit	Cell Signaling Technology	Cat# 2118 RRID:AB_561053
Anti-GFP mouse	Roche	Cat# 11814460001, RRID:AB_390913
Anti-Glucagon (D16G10) rabbit	Cell Signaling Technology	Cat# 2760 RRID:AB_659831
Anti-Insulin + Proinsulin mouse	Acris	Cat# AM20166PU-N RRID:AB_10696052
Anti-Insulin (C27C9) rabbit	Cell Signaling Technology	Cat# 3014 RRID:AB_2126503
Anti-Ki67-APC	Biolegend	Cat# 652405 RRID:AB_2561929
Anti-p16INK4A rabbit	Thermo Fisher Scientific	Cat# PA5-20379 RRID:AB_11157205
Anti-PKD/PKCμ (D4J1N) rabbit	Cell Signaling Technology	Cat# 90039 RRID:AB_2800149
Anti-Rabbit Alexa546 Fluor (H + L) IgG	Invitrogen	Cat# A-11035 RRID:AB_143051
Anti-Mouse IR Dye 800CW	LI-COR	Cat# 926-32210 RRID:AB_621842
Anti-rabbit IgG (H + L) HRP	Jackson Immunoresearch	Cat# 111-035-144 RRID:AB_2307391
Anti-mouse IgG (H + L) HRP	Jackson Immunoresearch	Cat# 115-035-062 RRID:AB_2338504
Chemicals and Peptides		
Collagenase P	Roche	Cat# 11213865001
CRT0066101	Selleckchem	Cat# S8366
Dextrose	Merck	Cat# D9434
Doxycycline	Yancheng Suhai Pharmaceutical	Cat# 137087-0008
Glucose	Roth	Cat# 6887.1
Insulin	Lilly	Cat# HI0210
Paraformaldehyde	Merck	Cat# P6148
Critical Commercial Assays		
CellROX™ Deep Red	Thermo Fisher Scientific	Cat# C10422
Insulin ELISA High Range	Alpco	Cat# 80-INSMSH-E01
Insulin ELISA Ultrasensitive	Alpco	Cat# 80-INSMSU-E01
Senescence β-Galactosidase Staining Kit	Cell Signaling Technology	Cat# 9860
Triglyceride Detection Kit	Thermo Fisher Scientific	Cat# TR22421
Experimental Models: Mouse-Strains		
ntg/+/−: Tg(Ins2-rtTA)2Efr/J; CD57BL/6	The Jackson Laboratory	Cat# 008250 RRID:IMSR_JAX:008250
PKDkd-EGFP: Tg(TetO-PKDkd-EGFP)/Tg(Ins2-rtTA)2Efr/J); CD57BL/6	University of Stuttgart, IZI	–
Tg(TetO-PKDkd-EGFP); CD57BL/6	University of Stuttgart, IZI	[28]
Wild-type: CD57BL/6	University of Stuttgart, IZI	–
Oligonucleotides (qPCR; F: Forward; R: Reverse)		
Actin	Qiagen	Cat# Mm_Actb_1_SG
SOD2 (MNSOD) F: 5′ GGCCAAGGGAGATGTTACAA 3′ R: 5′ GAACCTTGGACTCCCACA 3′	Microsynth	–
PKD1	Qiagen	Cat# Mm_Prkd1_1_SG
PKD2	Qiagen	Cat# Mm_Prkd2_1_SG
PKD3	Qiagen	Cat# Mm_Prkd3_1_SG
LAMP2A F: 5′ TGTATTTGGCTAATGGCTCAGC 3′ R: 5′ TATGGGCACAAGGAAGTTGTC 3′	Microsynth	–
p16 ^Ink4a^ F: 5′ CGCAGGTTCTTGGTCACTGT 3′ R: 5′ TGTTCACGAAAGCCAGAGCG 3′	Microsynth	–
Software		
FlowJo	FlowJo	RRID:SCR_008520
GraphPad Prism 10	GraphPad	RRID:SCR_002798
ImageJ	ImageJ	RRID:SCR_003070
ZEN (blue edition)	Zeiss	RRID:SCR_013672
Other		
0.9 % NaCl injection solution	Braun	Cat# 2246244
Low Fat Diet (LFD)	Altromin	Cat# C1090-10
High-fat diet (HFD)	Altromin	Cat# C1090-60
HBSS	Thermo Fisher Scientific	Cat# 14025092
NuPage Novex 4–12% Bis-Tris gels	Thermo Fisher Scientific	Cat# NP0336
PBS	Thermo Fisher Scientific	Cat# 70011036
RPMI-1640	Thermo Fisher Scientific	Cat# 11875093

### 4.2 Animal models

Animal experiments have been approved by state authorities and were carried out in accordance to federal guidelines. To generate Tg(TetO-PKDkd-EGFP)/Tg(Ins2-rtTA)2Efr/J) mice (called “PKDkd-EGFP” from now on), heterozygous Tg(Ins2-rtTA)2Efr/J mice (called “ntg/+/−“ from now on) were crossbred with heterozygous Tg(TetO-PKDkd-EGFP) mice.

For short-term *in vivo* and *ex vivo* (isolated islets) studies, 4-15 month-old PKDkd-EGFP and control (ntg/+/−) mice were continuously provided with drinking water supplemented with doxycycline (2 mg/ml, 5 % sucrose) for 7 days. On day 7, mice were subjected to either glucose tolerance test (GTT), insulin tolerance test (ITT) or were immediately euthanized for islet isolation. For short-term studies with the selective PKD inhibitor CRT0066101 4-6-month-old wild-type mice were treated with CRT0066101 (80 mg/kg; 5 % dextrose in 0.9 % NaCl) per os (p.o.) for 7 days, once daily, followed by GTT, ITT or islet isolation.

### 4.3 Glucose tolerance test

The mice were fasted over night before the GTT. Glucose levels were determined using a blood glucose meter (STADA Gluco Result, STADAPHARM GmbHand corresponding test stripes) by taking a blood drop from the tail vein. Blood glucose concentrations were measured before and 15, 30, 60, 90, 120 and 240 min after intraperitoneal (i.p.) glucose injection (2 g/kg body weight (bw) in 0.9 % NaCl; HFD studies: 1 g/kg bw in 0.9 % NaCl).

### 4.4 Insulin tolerance test

Mice were fasted for 6 h before the ITT. On the same day, glucose levels were determined before and 15, 30, 60 and 90 min after intraperitoneal (i.p.) insulin injection (0.75 IU/kg bw in 0.9 % NaCl) via blood sampling from the tail.

### 4.5 Islet isolation

Mice were euthanized, the abdominal cavity was opened and the bile duct as well as the gall bladder were localized. The small intestine was clamped to the right and to the left of the sphincter of Oddi. Then, a syringe (injection cannula: 30G, BD Microlance, Cat# 304000) was inserted into the gall bladder and moved down the bile duct, as close as possible towards the sphincter of Oddi. 2 ml collagenase P (1 mg/ml) were slowly injected into the pancreatic duct and the inflation of the pancreas was closely monitored. The inflated pancreas was removed and transferred into a 10 cm dish, where it was cut in small pieces with scissors. Next, 1 ml of collagenase P was added, and the entire solution was transferred to a 15 ml falcon. After a 10 min incubation at 37 °C, the digestion was stopped by adding 10 ml of ice-cold PBS. Further, the tissue pellet was washed twice with 10 ml PBS and dissolved in 15 ml HBSS. After filtering the solution through a 70 μm cell strainer (Falcon, Cat# 352350), islets were handpicked and transferred to a 3.5 cm petri dish with 5 ml HBSS. For *ex vivo* GSIS, islets were cultured overnight in 5 ml RPMI (10 % fetal calf serum (FCS) and 1 % penicillin/streptomycin (P/S)). For all other *ex vivo* studies, islets were processed on the same day.

### 4.6 Glucose stimulated insulin secretion

For *ex vivo* GSIS, 10 overnight-cultured (RPMI, 10 % FCS, 1 % P/S) islets per mouse were picked and transferred into 200 μl KBHB (120 mM NaCl, 4.7 mM KCl, 2.5 mM CaCl_2_, 1 mM KH_2_PO_4_, 1.2 mM MgSO_4_, 10 mM HEPES, 20 mM NaHCO_3_, 0.5 mg/ml BSA, pH 7.4) containing 5 mM glucose. All further steps were carried out in 200 μl KBHB. After 60 min of equilibration at 37 °C, the islets were transferred to 3 mM glucose for another 60 min to mimic starvation. Next, islets were transferred to 16 mM glucose for 60 min. The supernatants of 3 mM and 16 mM glucose incubation were collected and stored at −80 °C. Finally, the islets were transferred to 200 μl 1.5 % HCl in 70 % EtOH and stored at −80 °C. The insulin concentration was assessed via ELISA.

For *in vivo* GSIS, mice were starved for 16 h. The baseline glucose concentration was measured (blood glucose meter, via tail), glucose was injected i.p. (2 g/kg bw) and after 15 min the blood glucose concentration was analyzed. Additionally, blood was collected before and after the glucose challenge, to analyze the plasma insulin concentration via ELISA.

### 4.7 High-fat diet feeding

To induce obesity in PKD-EGFP and ntg/+/− mice, 2-month-old mice received either a high-fat diet (HFD, 35% fat) or a low-fat diet (LFD; control diet) for 16 weeks ad libidum and were continuously provided with doxycycline (200 μg/ml) supplemented drinking water. Blood was sampled via the tail once per week for triglyceride analysis. Body weight was monitored weekly. After 16 weeks, GTT, ITT or islet isolation were performed. For the HFD rescue experiment, 4-month-old wild-type CD57BL/6 mice received HFD during the first 16 weeks, followed by GTT or ITT. Afterwards, all mice received normal diet and were either treated p.o. with CRT0066101 (80 mg/kg; 5 % dextrose in 0.9 % NaCl; 5 days per week, once daily) or the carrier (5 % dextrose in 0.9 % NaCl; 5 days per week, once daily) during the weeks 17–19. Subsequently, ITT or GTT were performed.

### 4.8 Triglyceride assay

Triglyceride determination was conducted according to manufacturer's instructions (Thermo Fisher Scientific).

### 4.9 Insulin ELISA

To determine blood insulin concentrations during GTT plasma samples were directly measured using a commercially available insulin ELISA Kit. To determine insulin concentrations from *ex vivo* GSIS experiments, samples were diluted 1:5 (5 mM, 16 mM glucose) or 1:200 (total insulin content).

### 4.10 Reactive oxygen species quantification

Ten islets per mouse were transferred to 96-well plates and incubated for 10 min in 100 μl trypsin (1x) at 37 °C. Next, cells were singularized using a syringe (injection cannula: 27G) and washed once with PBA (2 % FCS, 0.2 % NaN_3_ in PBS). Cells were diluted in 200 μl PBA, the CellRox reagent (5 μM) was added, and plates were incubated for 30 min at 37 °C. Afterwards, cells were washed 3 times with PBA and analyzed via flow cytometry. Data was evaluated using FlowJo. Relative median fluorescence intensities (rel. MFI) were calculated with the following formula: rel. MFI = MFI_sample_/MFI_cells_.

### 4.11 Ki67 proliferation assay

Ten islets per mouse were transferred to 1.5 ml Eppendorf tubes and incubated for 10 min in 200 μl trypsin (1x) at 37 °C. Next, cells were singularized using a syringe (injection cannula: 27G) and washed once with PBS. For fixation, cells were resuspended in 300 μl ice-cold 70 % EtOH, vortexed and incubated on ice for 30 min. After a further washing step, cells were stained with Ki67-APC antibody for 30 min at 4 °C. Afterwards, cells were washed once and analyzed via flow cytometry. Data was evaluated using FlowJo. Data is presented as relative median fluorescence intensity (rel. MFI), rel. MFI = [MFI_sample_ – (MFI_isotype_ – MFI_cells_)]/MFI_cells_. The relative MFI of the two genotypes to be compared were normalized to the average value obtained for both genotypes.

### 4.12 Cell/islet size

For islet cell size, 10 islets per mouse were transferred to 96-well plates and incubated for 10 min in 100 μl trypsin (1x) at 37 °C. Next, cells were singularized using a syringe (injection cannula: 27G) and washed once with PBA (2 % FCS, 0.2 % NaN_3_ in PBS). Cells were resuspended and analyzed via flow cytometry. Data was evaluated using FlowJo. Data are presented as Median FSC-A. For islet area, 30 islets per genotype were imaged and the area was measured via ImageJ. For cryosectioning, whole pancreata were fixed in 4 % paraformaldehyde/PBS at 4 °C overnight, followed by incubation in 30 % sucrose/PBS at 4 °C for 24 h. Next, pancreata were mounted with cryostat embedding medium, frozen on dry ice and stored at −80 °C. Frozen pancreata were cut into 10–12 μm sections using a cryostat. Tissue sections were mounted on poly-lysine-coated microscope slides and stained for immunofluorescence microscopy. Images were obtained using a confocal laser scanning microscope. β-cell area was calculated by dividing the cross-section area of an islet by the number of nuclei.

### 4.13 Senescence β-Galactosidase staining

Five islets per mouse were transferred to 12-well plates and stained with the senescence β-galactosidase kit according to manufacturer's instructions overnight. Next day, pictures of the islets were taken with the Zeiss AXIO Zoom V16 microscope (objective ApoZ 1.5× 10.37 FWD 30 mm). The blue intensity of each islet was assessed using the ZEN software.

### 4.14 Quantitative real-time PCR

RNA was isolated from 50 islets per mouse, using the NucleoSpin® miRNA kit according to manufacturer's instructions. 10 ng RNA were used for real-time PCR, using the Power SYBR® Green RNA-to-C_T_™ 1-Step kit. Analysis was performed using the CFX96 Touch Real-Time PCR Detection System (Bio-RAD, 1855196). Expression levels of the target gene were normalized to those of the housekeeping gene (β-actin) using the ΔCt method.

### 4.15 Immunoblotting

Western blot analyses were conducted as previously reported [63]. In short, tissue was lysed, samples were loaded on NuPage Novex 4–12% Bis-Tris gels and proteins were blotted using the iBlot™ system (Thermo Fisher Scientific). Membranes were incubated with specific primary antibodies and proteins were visualized with HRP-tagged secondary antibodies or with IRdye-tagged secondary antibodies. Acquisition was done using X-ray films (Figure 1E), an Amersham™ Imager 600 device (Cytiva, Figure 5K) or the Odyssey Imager (LI-COR, Figure S1A).

### 4.16 Cryosectioning

Animals were euthanized using CO_2_ inhalation or sacrificed by cervical dislocation in accordance with institutional ethical guidelines. For pancreas dissection, the abdominal fur was soaked with 70% ethanol, and the abdominal cavity was opened using standard sterile procedures. The pancreas was identified and carefully excised. For brain preparation, a vertical midline scalp incision was made from the cervical-cranial junction to the nasal tip. Using a Liston bone-cutting forceps, the skull was opened and the brain was fully harvested. Freshly collected tissues were fixed in 4% paraformaldehyde (PFA) in PBS at 4 °C overnight. The following day, samples were transferred to 30% sucrose in PBS for cryoprotection and incubated at 4 °C for an additional 24 h. After cryoprotection, tissues were embedded in cryostat embedding medium and frozen on dry ice. Frozen tissues were cryosectioned at a thickness of 10–12 μm for pancreas and 30 μm for brain using a cryostat with both the box and object temperatures set to −20 °C. Sections were thaw-mounted onto polysine-coated microscope slides that had been pre-warmed to 55 °C. Prepared slides were stored at −80 °C until further processing.

### 4.17 Antigen retrieval

To break protein cross-links formed by formalin fixation and to unmask antigenic epitopes for immunohistochemistry, an optional antigen retrieval step was performed. Slides with cryostat sections were first thawed and air-dried at room temperature, followed by re-fixation in 4% paraformaldehyde (PFA) in PBS for 5 min at room temperature. Slides were then washed once with PBS (5 min, RT). For heat-induced antigen retrieval, sections were incubated in 10 mM sodium citrate buffer (pH 6.0) at 95 °C for 10 min. After cooling and drying, a hydrophobic barrier was applied around the tissue sections using a barrier pen. Sections were then permeabilized with 0.3% Triton X-100 in PBS for 5 min at room temperature and subsequently processed for immunostaining as described.

### 4.18 Immunofluorescence and microscopy

Cryostat sections were thawed at room temperature for 10–20 min, followed by drying and heat-fixation on a slide warmer at 75 °C for 10 min. A hydrophobic barrier was drawn around the tissue sections using a barrier pen. For immunofluorescence staining, slides were placed in a humidified chamber and subsequently fixed with 4% paraformaldehyde (PFA) in PBS for 5 min at room temperature. Following fixation, sections were washed twice with PBS for 5 min each and permeabilized with 0.3% Triton X-100 in PBS for 5 min at room temperature. Another set of two PBS washes (5 min each) was then performed. To block nonspecific binding, sections were incubated with 5% fetal calf serum (FCS) in PBS for 1 h at room temperature. Primary antibodies, diluted in PBS containing 5% FCS, were applied overnight at 4 °C. The next day, sections were washed twice with PBS (5 min each, room temperature) and incubated with appropriate fluorescently labeled secondary antibodies, also diluted in PBS/5% FCS, for 1.5 h at room temperature in the dark. After an additional two PBS washes (5 min each), sections were mounted using Mowiol mounting medium containing DAPI and allowed to dry overnight in the dark. Isolation and staining of islets was performed according to [64]. Imaging of islets and pancreas sections was performed using a confocal laser scanning microscope (LSM710, Carl Zeiss Microscopy, Germany).The following objectives were used: Plan-Apochromat 63x/1.4 oil, Plan-Apochromat 40x/1.3 oil, Plan-Apochromat 20x/0.8 air. DAPI was excited with a 405 nm diode laser, its emission was detected from 410 nm to 495 nm. EGFP was excited with the 488 nm line of an argon laser, its emission was detected from 496 nm to 553 nm. Alexa 546 was excited with a 561 nm DPSS laser and its emission was detected from 563 nm to 620 nm. EGFP channel images were processed with a Gaussian filter using a value of 1.05 for Sigma X and Sigma Y. Brain sections were imaged at a CellObserver Z.1 (Carl Zeiss Microscopy, Germany) using an EC-Plan Neofluar 10x/0.3 objective, a Colibri1 LED excitation light source (DAPI: 365 nm, EGFP: 470 nm) in combination with Zeiss Filterset 62 and an Axiocam Mrm camera. Single images were acquired with 10 % overlap and stitched afterwards to a tile region using Zen blue software version 2.3 (Carl Zeiss microscopy).

### 4.19 Statistical analysis

Prior to statistical analysis, data were tested for normality using the D'Agostino–Pearson omnibus test or the Shapiro–Wilk test. Comparisons between multiple groups were performed using ordinary one-way or two-way ANOVA (for normally distributed data) or the Kruskal–Wallis test (for non-parametric data), followed by appropriate post hoc tests for multiple comparisons. Comparisons between two groups were performed using unpaired, two-tailed Student's t-tests. Data was analyzed using GraphPad Prism 10. P-values: P ≤ 0.05 (∗), P ≤ 0.01 (∗∗), P ≤ 0.001 (∗∗∗), P ≤ 0.0001 (∗∗∗∗). Only significant differences are shown.

## CRediT authorship contribution statement

**Wolfgang S. Lieb:** Writing – original draft, Visualization, Methodology, Investigation, Formal analysis, Conceptualization. **Carlos O. Oueslati Morales:** Writing – original draft, Investigation. **Kornelia Ellwanger:** Supervision, Methodology, Investigation. **Claudia Koch:** Methodology, Investigation, Formal analysis. **Sylke Lutz:** Methodology, Investigation. **Stephan A. Eisler:** Visualization. **Annika M. Möller:** Formal analysis, Visualization. **Veronika Leiss:** Writing – original draft, Methodology. **Angelika Hausser:** Writing – original draft, Writing - review & editing, Visualization, Supervision, Funding acquisition, Formal analysis, Conceptualization.

## Funding

Work in the lab of Angelika Hausser is supported by grants from the Deutsche Forschungsgemeinschaft (DFG) (HA-3557/11-2 and 11-3).

## Declaration of competing interest

The authors declare that they have no known competing financial interests or personal relationships that could have appeared to influence the work reported in this paper.

## Figures and Tables

**figs3:**
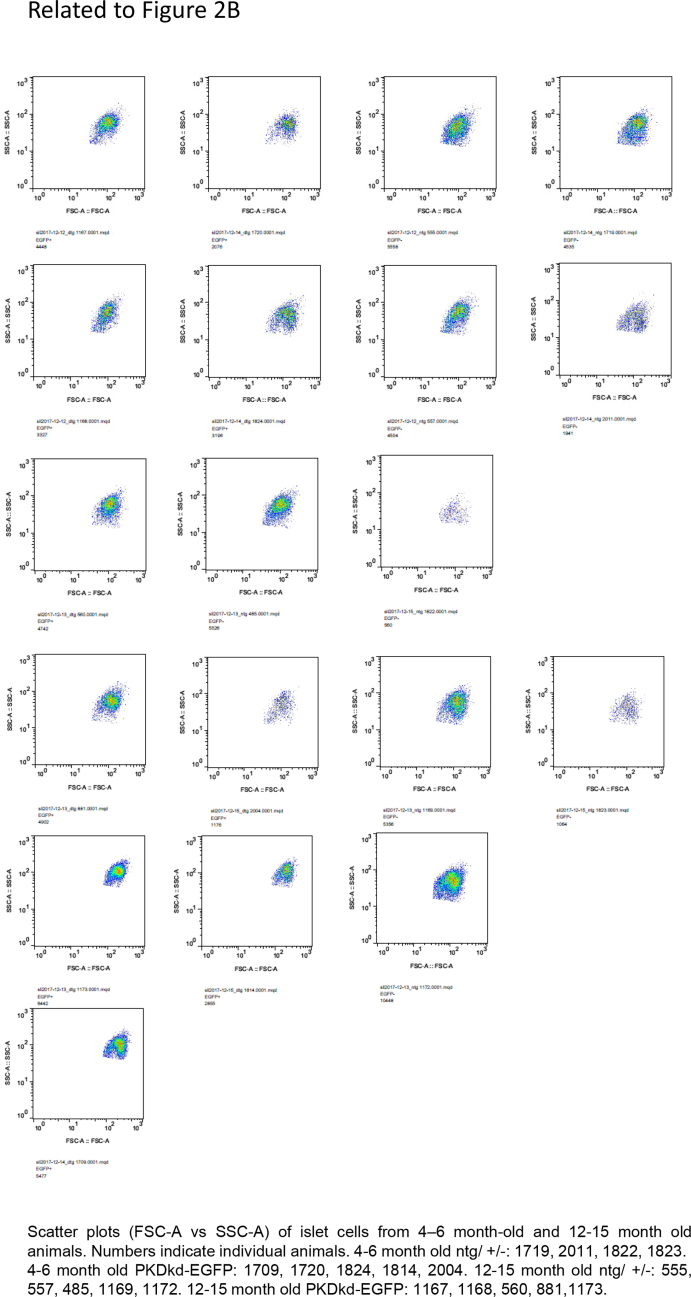


**figs4:**
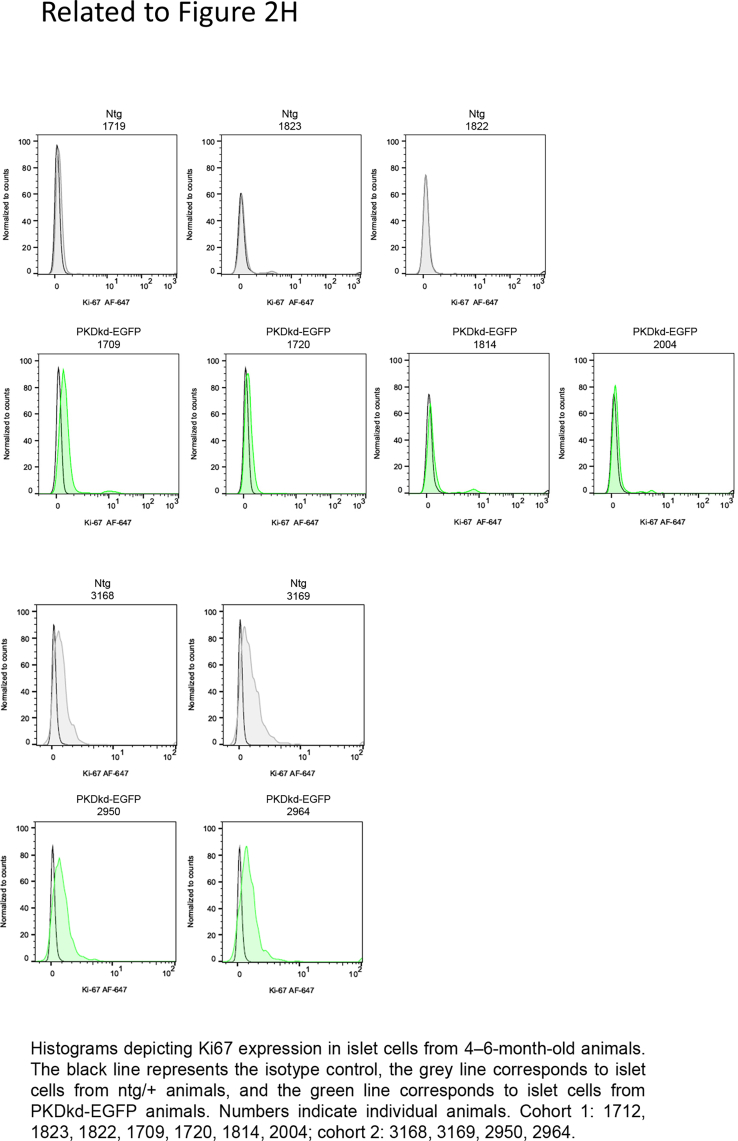


## Data Availability

Data will be made available on request.
