# Supplementary figures and images for: Protein kinase D deficiency induces a senescence-like phenotype in β-cells and improves glucose and insulin tolerance under high-fat diet conditions
Source: Mol Metab. 2025 Dec 3;103:102297. doi: 10.1016/j.molmet.2025.102297 (PMC12775600; doi:10.1016/j.molmet.2025.102297)

Figure 1E

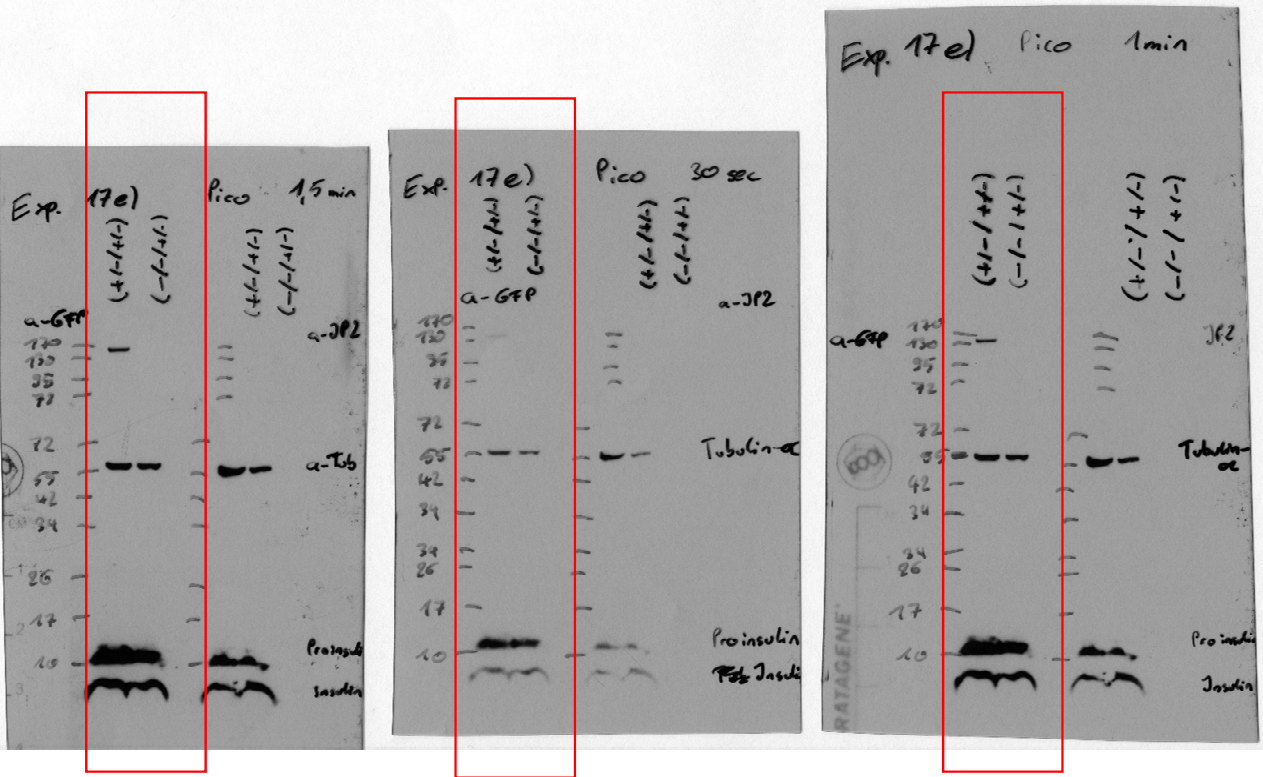

Figure 5K

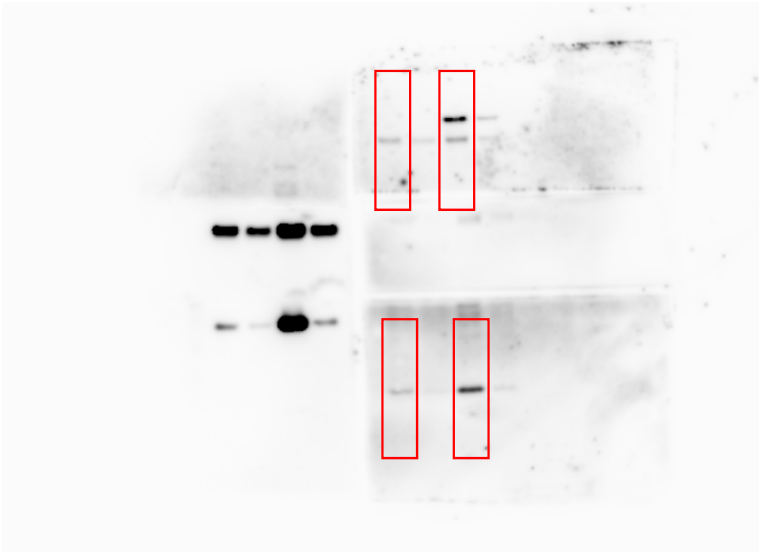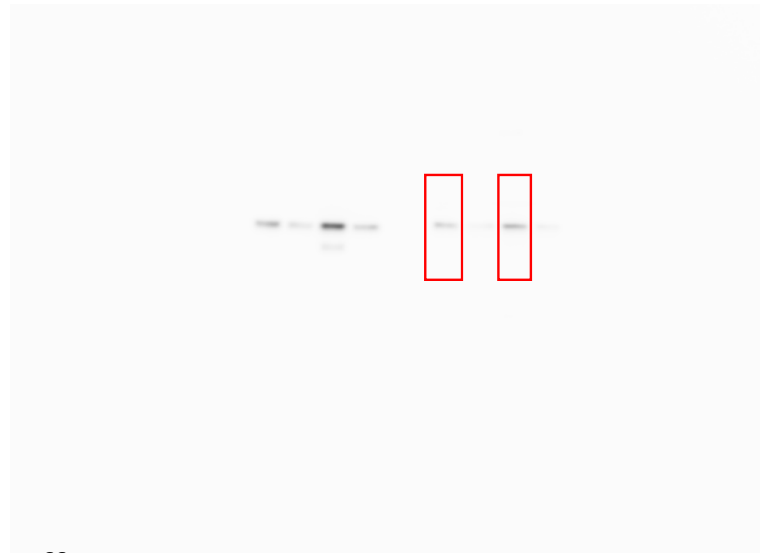

tiff

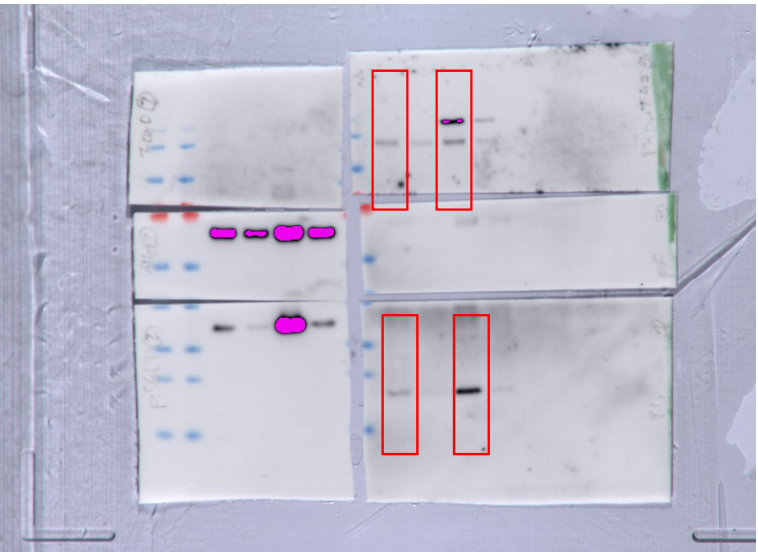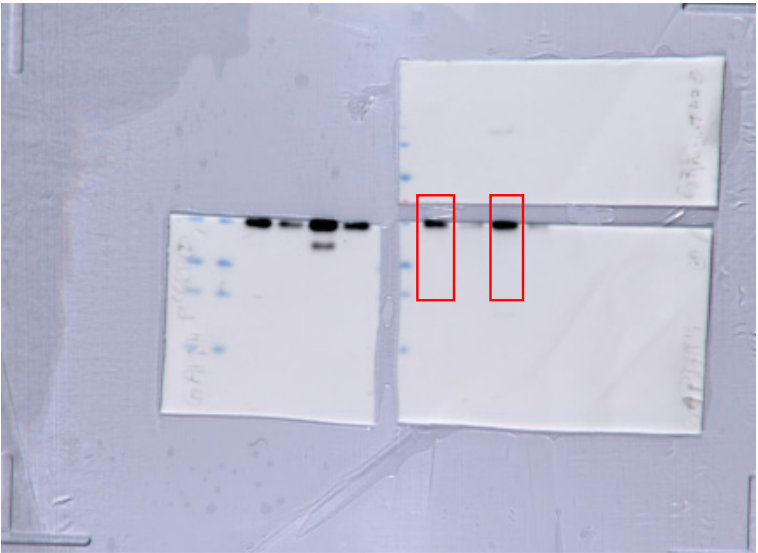

jpg

PKD

p16

GAPDH

Figure S1A, original LI-COR image

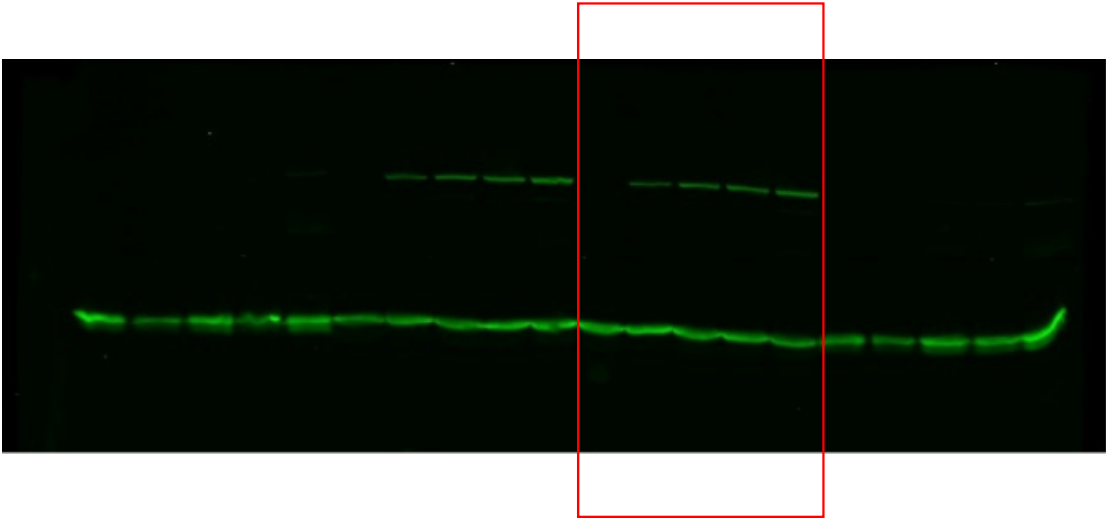

GFP

Alpha-tubulin

Supplement: Multimedia component 1 [file mmc1.pdf]
